# Humoral immunogenicity and reactogenicity of CoronaVac or ZF2001 booster after two doses of inactivated vaccine

**DOI:** 10.1038/s41422-021-00596-5

**Published:** 2021-12-03

**Authors:** Yunlong Cao, Xiaohua Hao, Xi Wang, Qianhui Wu, Rui Song, Dong Zhao, Weiliang Song, Yao Wang, Ayijiang Yisimayi, Wei Wang, Wen Zhang, Juan Du, Hongjie Yu, Xiaoliang Sunney Xie, Ronghua Jin

**Affiliations:** 1 Changping Laboratory, Beijing, China; 2 Biomedical Pioneering Innovation Center (BIOPIC), Peking University, Beijing, China; 3 Beijing Advanced Innovation Center for Genomics (ICG), Peking University, Beijing, China; 4 National Center For Infectious Diseases, Beijing Ditan Hospital, Capital Medical University, Beijing, China; 5 Beijing Key Laboratory of Emerging Infectious Diseases, Institute of Infectious Diseases, Beijing Ditan Hospital, Capital Medical University, Beijing, China; 6 School of Public Health, Fudan University, Key Laboratory of Public Health Safety, Ministry of Education, Shanghai, China; 7 School of Life Sciences, Peking University, Beijing, China; 8 Shanghai Institute of Infectious Disease and Biosecurity, Fudan University, Shanghai, China; 9 Department of Infectious Diseases, Huashan Hospital, Fudan University, Shanghai, China

**Keywords:** Immunology, Molecular biology

Dear Editor,

COVID-19 vaccination campaigns are being conducted in countries worldwide, and 47.4% of the world population has received at least one dose of a COVID-19 vaccine.^1^ Although vaccination has shaped COVID-19 epidemic curves, waning antibody levels and relatively short-duration protection provided by current COVID-19 vaccines have been observed, especially against SARS-CoV-2 variants of concern (VOCs) and among older individuals.^2^ Booster dose programs have been started in nearly 50 countries, and preliminary evaluation shows that the additional doses reduce breakthrough infections and numbers of symptomatic cases.^1,3^ The World Health Organization now recommends that for Sinovac and Sinopharm inactivated vaccines, immunization programs should offer an additional (third) dose of the homologous vaccine for people 60 years and older as part of an extended primary series, and that heterologous platforms vaccine for the additional dose may also be considered based on vaccine supply and access considerations. Interim results from heterologous prime-boost studies showed that boosting with heterologous platform vaccines could induce significantly higher titers of neutralizing antibodies and better cellular immunity, providing evidence for programmatic consideration of an alternative to homologous boosting.^4^

Previous studies showed that ZF2001, an RBD-subunit vaccine, could induce humoral immunity that exhibits better tolerance to current VOCs compared to inactivated vaccines and natural infections, suggesting ZF2001 as an ideal candidate for heterologous booster.^5^ To assess the impact of a heterologous third dose of ZF2001 or a homologous third dose of CoronaVac on vaccine-induced antibodies against VOCs, we conducted a single-center, open-label, randomized controlled clinical trial among healthcare professionals at Beijing Ditan Hospital who had received two doses of CoronaVac in a 28-day interval 4–8 months earlier. Eligible participants were randomly assigned to receive either one dose of CoronaVac or ZF2001 vaccine or no intervention (1:2:1). Specifically, 164 participants were enrolled and randomly assigned to receive either CoronaVac (*n* = 41), ZF2001 (*n* = 81) or no vaccine (*n* = 42). 163 participants were included in the immunogenicity analyses (CoronaVac 41, ZF2001 81, Control 41), and 122 were included in the safety analyses (CoronaVac 41, ZF2001 81) (Supplementary information, Fig. S1). The mean ages of the three groups were 38.1 (Standard Deviation (SD) = 10.90), 40.7 (SD = 8.70), 37.1 (SD = 8.05) years old, respectively. Baseline characteristics were similar among groups (Supplementary information, Table S1). We assessed the SARS-CoV-2 anti-spike IgG antibody levels and the geometric mean titers (GMTs) against authentic prototype SARS-CoV-2 and Beta, Gamma, and Delta variants on day 0 and 14 after administration of third doses for those vaccinated and for control group subjects. 35 COVID-19 human convalescent sera (HCS) donated 30–40 days since onset were also accessed for comparison.

At 4–8 months after primary immunization with CoronaVac, neutralizing antibody levels against the three variants are close to the lower limit of detection (8-fold dilution of plasma) (Fig. 1). The third dose of either CoronaVac or ZF2001 vaccine rapidly induced a significantly high degree of humoral immunogenicity; the humoral immune response induced by ZF2001 was higher than that from CoronaVac (Fig. 1; Supplementary information, Figs. S2, S3). GMTs in all groups were higher against the prototype strain than against Gamma, Beta, and Delta variants (Supplementary information, Fig. S4). In the CoronaVac group, GMTs assessed by pairwise comparison increased from 34 to 794 against prototype (23.3-fold), from 7 to 123 against Beta (18.6-fold), from 7 to 162 against Gamma (23.8-fold), and from 5 to 86 against Delta (18.4-fold), in consistence with recent CoronaVac booster studies.^6^ In the ZF2001 group, GMTs increased from 39 to 1306 against prototype (33.9-fold), from 7 to 301 against Beta (44.5-fold), from 8 to 274 against Gamma (32.7-fold), and from 5 to 205 against Delta (39.1-fold). As for the control group, neutralization titers showed no significant changes against all four SARS-CoV-2 strains during the 14-day follow-up. Seroconversion rates 14 days after third doses, which was defined as a change of titers from seronegative at baseline to seropositive, or a four-fold increase of titers for individuals whose titers at day 0 were above seropositive cutoffs (8-fold dilution of plasma), were all above 90% in both vaccinated groups (Supplementary information, Table S2).

**Fig. 1:**
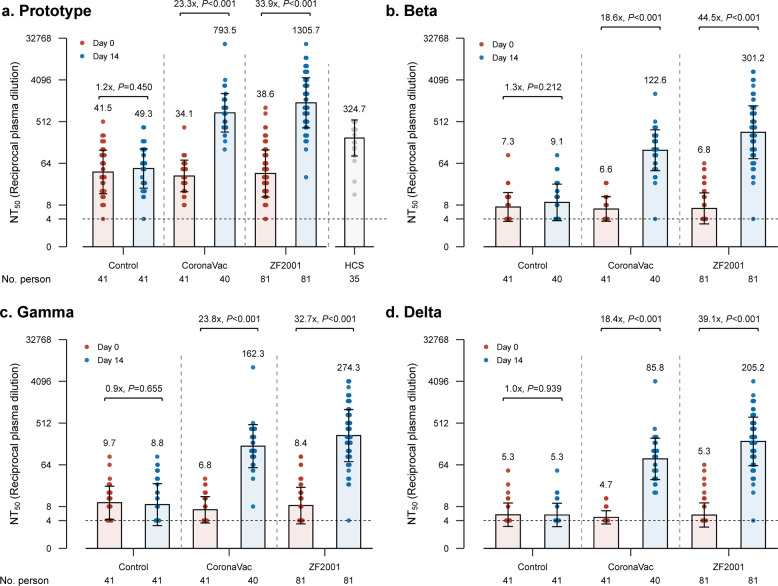
Humoral immune responses against prototype and variants of SARS-CoV-2 among participants. Results of authentic virus neutralization assays using participants’ plasma against SARS-CoV-2 prototype strain (**a**), Beta strain (**b**), Gamma strain (**c**), and Delta strain (**d**). The geometric mean titer (GMT), geometric standard deviation, and pairwise fold-changes of 50% neutralization titers are labeled. HCS, human convalescent serum. Booster doses were administrated on Day 0. Statistical significances were analyzed by two-sided paired t-tests with log-transformation.

ZF2001 RBD-subunit vaccine may induce and enrich RBD-directed neutralizing antibodies that are more resistant to SARS-CoV-2 mutations due to their diverse modes of RBD binding, especially when compared to N-terminal domain (NTD) neutralizing antibodies which were mostly escaped by mutations on the NTD antigenic supersite.^5^ Indeed, the GMT fold-change difference between ZF2001 and CoronaVac booster dose is higher against Delta and Beta strains than prototype and Gamma strains, where the former two carry NTD deletions that abolish the efficacy of NTD neutralizing antibodies elicited by CoronaVac (Supplementary information, Fig. S2). Also, SARS-CoV-2 anti-spike IgG antibody titers were significantly higher in participants boosted with ZF2001 than in participants boosted with CoronaVac (Supplementary information, Fig. S3). Together, we demonstrated that the ZF2001 third dose boost should be more efficient in terms of humoral immunity against SARS-CoV-2 variants, especially those harboring NTD antigenic supersite mutations, such as the Delta strain.

Additionally, we conducted subgroup analyses in the intervention groups. At 14 days after administration of CoronaVac booster, there were no differences in the humoral response to the four strains by gender, but interestingly, the responses were greater in female recipients 14 days after ZF2001 (Supplementary information, Fig. S5). Differences in humoral immunologic response against the four SARS-CoV-2 strains did not vary by age group (over or under 40 years of age) (Supplementary information, Fig. S6). There were no differences in antibody level increases by vaccine type, regardless of whether the interval between the second dose and the third dose was 4–5 months or 6–8 months (Supplementary information, Figs. S7–S9). GMTs and corresponding 95% confidence intervals (CIs) against authentic SARS-CoV-2 variants, and by age, gender, and second-to-third dose interval are shown in Supplementary information, Table S3.

Importantly, both of the three-dose regimens were well tolerated. The most common adverse reactions were local injection site reactions, and all adverse reactions were grade 1. The overall incidence of adverse reactions within 14 days after the third dose was 39.0% (16 of 41 participants) in the CoronaVac group and 23.5% (19 of 81) in the ZF2001 group; most occurred within 24 h of administration. There was no significant difference in any adverse reaction by vaccine type (Supplementary information, Table S4). The proportion of adverse events for the heterologous ZF2001 booster group was similar to the results obtained in phase 2 clinical trials of ZF2001 (40/148, 27.0%, *P* = 0.67), suggesting no increase in the proportion of side effects for the ZF2001 booster dose.^7^ The adverse events rate for the homologous CoronaVac booster group was higher than the results obtained in the two-dose CoronaVac clinical trial (Phase II, 26/144, 18.1%, *P* = 0.009) and the third-dose booster clinical trial (Phase II, 10/52, *P* = 0.06);^8,9^ however, all adverse reactions were grade 1, and the higher proportion of adverse reactions may be caused by the small sample size. Together, these results suggest that booster doses were well tolerated in adult participants during the short-term follow-up period. Long-term population-level surveillance is needed to further augment the safety profile of booster doses.

In the current pandemic situation with the Delta variant being predominant, analysis of the neutralizing capacity of vaccines against SARS-CoV-2 variants is of utmost relevance. Since CoronaVac accounts for almost a quarter of the COVID-19 vaccine doses delivered globally, our results have broad applicability as they help understand the potential impact of booster vaccinations, especially for those based on 2-dose CoronaVac. Early estimates of the effectiveness of booster doses in Chile showed that homologous CoronaVac booster could increase the effectiveness against COVID-19 from 56% to 80%.^10^ Evidence of improvement of protection from symptomatic illness afforded by a booster dose in Chile when the Delta variant was circulating suggests that homologous booster vaccination of CoronaVac is a good candidate to curb the pandemic in the real world. Here, our results suggest that heterologous boosters, such as RBD subunit vaccines, may also contribute to the protection from SARS-CoV-2 symptomatic illness, and may be more efficient against VOCs. It is notable that participants in our study were young adults, and therefore our results do not necessarily extend to different risk groups such as immunosuppressed individuals or elderly populations. Booster dose effectiveness for these high-risk groups needs to be assessed in multi-center studies with larger sample sizes.

## Supplementary information

Supplementary Information
