# Supplementary material for: Humoral immunogenicity and reactogenicity of CoronaVac or ZF2001 booster after two doses of inactivated vaccine
Source: Cell Res. 2021 Dec 3;32(1):107–9. doi: 10.1038/s41422-021-00596-5 (PMC8640508; doi:10.1038/s41422-021-00596-5)
Supplement: Supplementary file 1 — Supplementary Information [file 41422_2021_596_MOESM1_ESM.pdf]

Supplementary Information

Materials and Methods

Study protocol

The study protocol and informed consent form were approved by the Ethics Committee of Beijing Ditan Hospital Capital Medical University (IRB#2021-(024)-02). Written informed consent was obtained before the enrollment. This trial was registered with ChiCTR2100051998.

Study design

This study was a single-center, open-label, randomized controlled clinical trial initiated in Beijing Ditan Hospital, China, on August 7 2021, among healthy healthcare workers. Subjects who received 2 doses of CoronaVac at 28-day intervals 4-8 months ago and voluntarily received the third dose of vaccine (CoronaVac or ZF2001) were eligible for this study. Randomization codes were generated individually, and participants were randomly assigned using block randomization with a block size of eight, developed with SAS software (version 9.4). Eligible participants were assigned sequentially to receive one dose of CoronaVac or ZF2001 or no intervention (1:2:1).

Inclusion criteria

1) Adult subjects (18 years old) having received two doses of inactivated COVID-19 vaccines between 4 and 8 months before the screening visit.

2) Subjects were in good health evaluated by investigators by inquiring case history

and physical examination.

3) Female subjects of childbearing age have no lactation or pregnancy (negative urine pregnancy test) at the time of enrollment or no plan for family within the first 3 months after enrollment, take effective contraceptive measures within 2 weeks before enrollment.

4) Participants must provide consent indicating that he or she understands the purpose, procedures and potential risks and benefits of the study, and is willing to participate in the study and adhere to the procedures specified in the protocol.

Exclusion criteria

1) Positive results for RT-qPCR tests of SARS-CoV-2.

2) Participant has clinical symptoms including fever, hoose, fatigue, stuffy nose, runny nose, sore throat, myalgia, diarrhea, shortness of breath, dyspnea, etc.

3) temperature ≥37.0℃ prior to vaccination.

4) Positive results for the urine pregnancy test.

5) Participant has a known or suspected allergy or history of anaphylaxis or other serious adverse reactions to inactivated vaccine excipients

6) Participant has a history of convulsions, epilepsy, encephalopathy or mental illness or family history;

7) Participant has illness including congenital malformations or developmental disorders, genetic defects, severe malnutrition, etc.;

8) Participant has severe liver and kidney disease and uncontrollable hypertension

(systolic blood pressure >= 140 mmHg, diastolic blood pressure >= 90 mmHg), diabetes complications, malignant tumors, acute or chronic diseases.

9) Participant was diagnosed with congenital or acquired immunodeficiency, HIV infection, lymphoma, leukemia, or other autoimmune diseases;

10) Participant was receiving anti-tuberculosis treatment;

11) Participant has known or suspected diseases including severe respiratory diseases, severe cardiovascular diseases, liver and kidney diseases, and malignant tumors;

12) Participant had a history of coagulation dysfunction (such as coagulation factor deficiency and coagulation diseases);

13) Participant received immunotherapy or inhibitor treatment within 3 months before enrollment (continuous oral or infusion for more than 14 days);

14) Participant received live attenuated vaccine within 1 month or other vaccines within 14 days before enrollment;

15) Participant received other study drugs within 3 months before enrollment;

16) Any condition or situation precluding or interfering the compliance with the protocol assessed by investigators.

Assessment of Safety

Solicited local and systemic adverse reactions were collected by investigators on each visit. The severity of adverse reactions was graded according to Common Terminology Criteria for Adverse Events (version 5.0). The existence of causal associations between adverse events and vaccination was determined by the investigators.

Outcomes

The primary outcome was seroconversion rate on day 14 after the third dose of neutralizing antibodies to authentic SARS-CoV-2, which was defined as a change of titers from seronegative at baseline to seropositive, or a four-fold increase of titers for individuals whose titers were above seropositive cutoffs (1:8). Secondary immunological outcomes included geometric mean titers (GMTs) of neutralizing antibodies to infectious SARS-CoV-2 and pre-immunization (baseline) and on day 14 after third doses. Blood samples of participants in the control group were taken on the same day along with intervention groups. Safety outcomes were incidences and severity of adverse events occurred within 14 days after the third dose, as well as the association with the injection evaluated by investigators.

Convalescent serum samples

Human convalescent serum (HCS) samples were obtained from 35 people who had had a laboratory-confirmed COVID-19 diagnosis and had been recovered for at least 2 weeks as control. Serum samples were collected between 30-40 days since onset. The severity of COVID-19 illness was all classified as mild-to-moderate. Most of the participants were females (n=22, 62.9%). The mean age among participants were 45.4 (SD 16.8) years.

Plasma collection

Whole blood samples were subjected to Ficoll (Cytiva, 17-1440-03) gradient centrifugation after 1:1 dilution in PBS (Invitrogen, C10010500BT) + 2% FBS (Gibco, A3160901). After centrifugation, plasma was collected from the upper layer.

Authentic virus neutralization

Authentic SARS-CoV-2 neutralization experiments were performed using CPE assay. Serum/plasma samples were inactivated at 56℃ for 0.5h and serially diluted with cell culture medium in two-fold steps. The diluted plasma was mixed with a virus suspension of $100TCID_{50}$ in 96-well plates at a ratio of 1:1, followed by 2 hours incubation at 36.5°C in a 5% CO2 incubator. $1x10^4$ Vero cells (ATCC, CCL-81) were then added to the serum-virus mixture, and the plates were incubated for 5 days at 36.5°C in a 5% CO2 incubator. The cytopathic effect (CPE) of each well was recorded under microscopes. Assays for each sample were replicated once and the neutralization titer was calculated by the Spearman-Karber method accounting for the previous 2-fold dilution during plasma collection. All experiments were performed in a Biosafety Level 3 laboratory.

ELISA

ELISA plates were coated with 100 μL 1 μg/mL antigen protein (SARS-CoV-2 Spike protein) overnight (16~20 hours) at 2~8℃. Following three times of washing and blocking with 10% FBS in 0.01M PBS at 37℃ for 120 min, serially diluted serum samples were added to each well and incubated at 37℃ for 60 min. Then plates were

washed three times and incubated with anti-IgG at 37℃ for 60 min. After five times of washing, substrate solution was added to each well and incubated for 15 minutes at room temperature, and then the stop solution buffer was added. Absorbance at 450 nm was measured. The positive sample is defined as the sample OD value ≥ 2.1 times of negative serum OD value. Endpoint titer was defined using the highest positive dilution-fold.

Statistical analysis

We assessed immunogenic endpoints at individual-level and group-level, respectively, which included participants who had both antibody results available according to the protocol at two visits and who had antibody results available at either of the two visits. Safety assessment was performed in a safety population data set of all subjects who received the third dose.

The demographics of participants were summarized for intervention groups. Pearson χ^2 test or Fisher's exact test was used to analyze categorical outcomes. GMTs and corresponding 95% CIs were calculated based on the standard normal distribution of log-transformed antibody titers. Seroconversion rate and corresponding 95% CIs were derived from a binomial distribution. Pairwise comparisons were conducted by group t-tests, nonparametric Kruskal-Wallis H test, and Wilcoxon signed-rank test with log-transformation. Hypothesis testing was two-sided, and we considered p values of less than 0.05 to be significant. We used R software version 4.1.0 for all analyses.

Table S1. Baseline characteristics

	CoronaVac group (N=41)	ZF2001 group (N=81)	Control group (N=42)
Sex			
Male	10 (24.4%)	25 (30.9%)	12 (28.6%)
Female	31 (75.6%)	56 (69.1%)	30 (71.4%)
Age, years			
Mean (SD)	38.1 (10.90)	40.7 (8.70)	37.1 (8.05)
Age groups			
18-39 years	25/41 (61.0%)	39/81 (48.1%)	27/42 (64.3%)
Male	7/25 (28.0%)	11/39 (28.2%)	7/27 (25.9%)
Female	18/25 (72.0%)	28/39 (71.8%)	20/27 (74.1%)
≥40 years	16/41 (39.0%)	42/81 (51.9%)	15/42 (35.7%)
Male	3/16 (18.8%)	14/42 (33.3%)	5/15 (33.3%)
Female	13/16 (81.2%)	28/42 (66.7%)	10/15 (66.7%)
Interval between second and third doses (months)			
4-5	17/41 (41.5%)	36/81 (44.4%)	—
6-8	24/41 (58.5%)	45/81 (55.6%)	—

Table S2. Estimated seroconversion rate on day 14 after third doses

Strain	CoronaVac group			ZF2001 group		
	**Total no. of participants**	**No. of seroconverted**	**Seroconversion rate (%, 95% CI)**	**Total no. of participants**	**No. of seroconverted**	**Seroconversion rate (%, 95% CI)**
Prototype	40	39	97.5 (86.8, 99.9)	81	73	90.1 (81.5, 95.6)
Beta	40	38	95.0 (83.1, 99.4)	81	79	97.5 (91.4, 99.7)
Gamma	40	39	97.5 (86.8, 99.9)	81	79	97.5 (91.4, 99.7)
Delta	40	40	100 (91.2, 100)	81	78	96.3 (89.6, 99.2)

Table S3. Neuralization antibody titers against authentic SARS-CoV-2 among all participants in vaccinated groups (GMT, 95%CI).

Characteristics	Group	No. of person	Day 0				Day 14			
			Prototype	Beta	Gamma	Delta	Prototype	Beta	Gamma	Delta
Total	CoronaVac	41	34 (27, 43)	7 (5, 8)	7 (6, 8)	5 (4, 5)	794 (589, 1069)	123 (89, 168)	162 (116, 226)	86 (62, 118)
	ZF2001	81	39 (30, 50)	7 (6, 8)	8 (7, 10)	5 (5, 6)	1306 (995, 1713)	301 (226, 401)	274 (207, 364)	205 (158, 267)
Age (years)										
<40	CoronaVac	25	33 (25, 43)	7 (5, 9)	7 (5, 9)	5 (4, 6)	830 (558, 1235)	145 (101, 210)	198 (130, 302)	97 (62, 151)
	ZF2001	39	43 (30, 63)	7 (6, 9)	8 (6, 11)	5 (4, 7)	1388 (953, 2021)	312 (206, 473)	279 (182, 427)	223 (156, 317)
≥40	CoronaVac	16	36 (23, 57)	6 (5, 8)	6 (5, 9)	4 (4, 5)	736 (468, 1157)	92 (52, 163)	116 (69, 195)	71 (46, 108)
	ZF2001	42	35 (24, 49)	7 (5, 8)	9 (6, 11)	5 (4, 6)	1234 (832, 1830)	292 (195, 435)	270 (185, 395)	190 (129, 281)
Gender										
Male	CoronaVac	10	42 (28, 63)	6 (4, 8)	8 (5, 13)	5 (4, 6)	950 (559, 1616)	151 (94, 241)	209 (135, 326)	112 (65, 192)
	ZF2001	25	24 (14, 39)	6 (4, 8)	6 (4, 8)	5 (4, 6)	690 (473, 1008)	161 (104, 248)	145 (94, 225)	115 (77, 172)
Female	CoronaVac	31	32 (24, 43)	7 (6, 9)	6 (5, 8)	5 (4, 5)	747 (523, 1068)	115 (77, 170)	149 (98, 226)	79 (53, 115)
	ZF2001	56	48 (36, 63)	7 (6, 9)	10 (8, 12)	6 (5, 7)	1736 (1248, 2414)	399 (283, 563)	364 (260, 510)	266 (194, 364)
Interval between second and third doses (months)										
4-5	CoronaVac	17	34 (24, 47)	7 (5, 10)	7 (5, 10)	5 (4, 5)	873 (574, 1329)	131 (87, 195)	181 (124, 262)	81 (53, 124)
	ZF2001	36	41 (28, 59)	6 (5, 7)	8 (6, 11)	5 (4, 6)	1611 (1072, 2421)	360 (246, 527)	331 (221, 496)	238 (161, 350)
6-8	CoronaVac	24	34 (24, 49)	6 (5, 8)	7 (5, 9)	5 (4, 6)	739 (486, 1125)	117 (73, 187)	150 (90, 251)	89 (56, 142)
	ZF2001	45	37 (26, 53)	8 (6, 10)	9 (6, 11)	5 (5, 6)	1104 (769, 1584)	261 (172, 395)	236 (159, 349)	183 (128, 261)

Table S4. Safety profile

	CoronaVac (N=41)	ZF2001 (N=81)	P value
Total	16 (39.0%)	19 (23.5%)	0.11
Within 24 hours			
Any adverse reaction	13 (31.7%)	14 (16.7%)	0.11
Any local reaction	12 (29.3%)	14 (16.7%)	0.20
Injection local pain	10 (24.4%)	13 (16.0%)	0.39
Induration	3 (7.3%)	1 (1.2%)	0.11
Redness	3 (7.3%)	1 (1.2%)	0.11
Swelling	3 (7.3%)	0 (0.0%)	0.04
Itch	3 (7.3%)	1 (1.2%)	0.11
Any systemic reaction	1 (2.4%)	1 (1.2%)	1.00
Fatigue	1 (2.4%)	0 (0.0%)	0.34
Dizziness	0 (0.0%)	1 (1.2%)	1.00
Day 1-14			
Any adverse reaction	4 (9.8%)	7 (8.6%)	1.00
Any local reaction	2 (4.9%)	4 (4.9%)	1.00
Injection local pain	2 (4.9%)	4 (4.9%)	1.00
Induration	1 (2.4%)	0 (0.0%)	0.34
Any systemic reaction	2 (4.9%)	5 (6.2%)	1.00
Fatigue	0 (0.0%)	3 (3.7%)	0.55
Abdominal discomfort	1 (2.4%)	0 (0.0%)	0.34

Menstrual delay	1 (2.4%)	0 (0.0%)	0.34
Pruritus	0 (0.0%)	1 (1.2%)	1.00
Sleepy	0 (0.0%)	1 (1.2%)	1.00

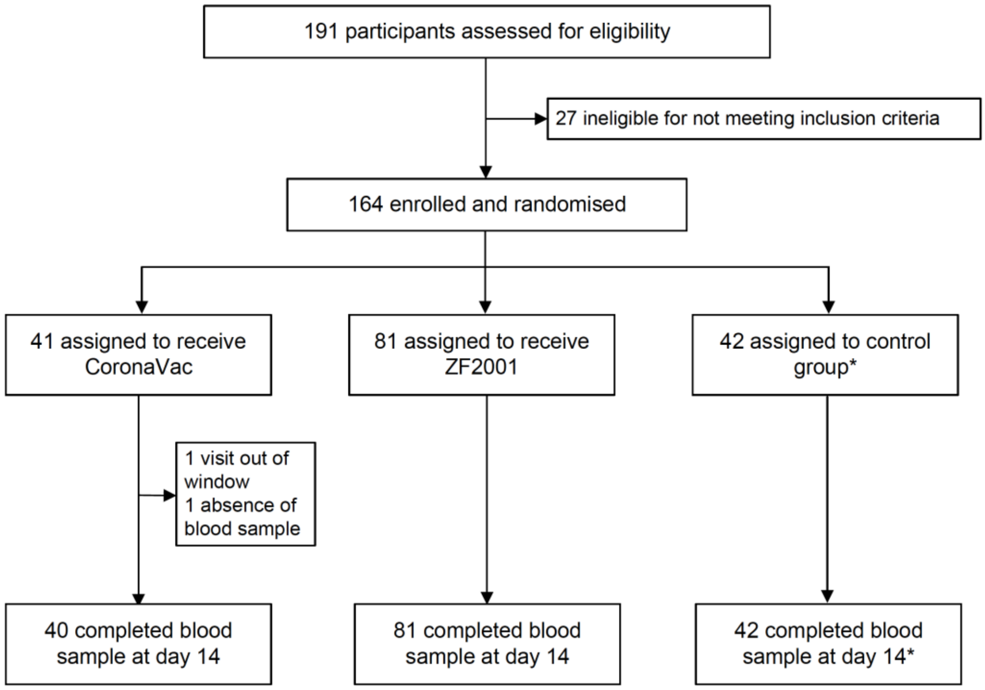

Fig. S1. Design of the clinical trial.

*Indicates hemolysis in blood samples taken from one participant in the control group on day 0 and 14, resulting in the lack of neutralization results.

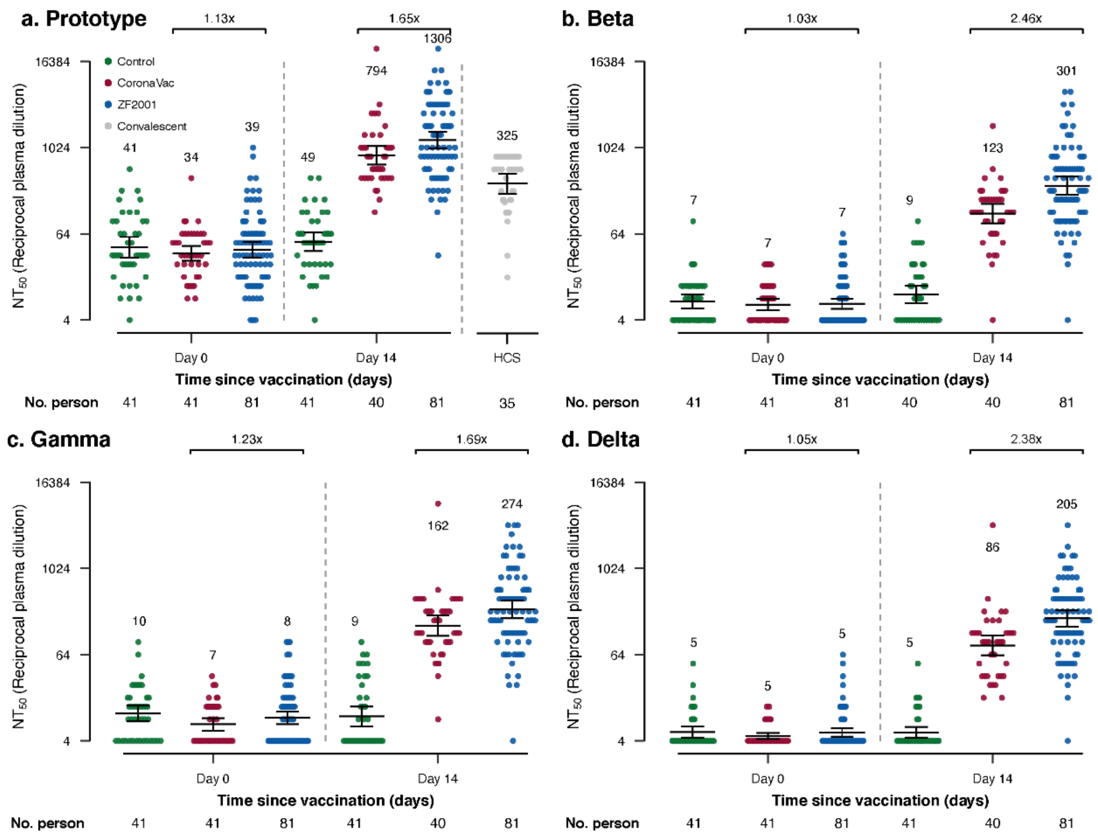

Fig. S2. Humoral immune responses comparison between homologous and heterologous boosters.

Comparison of authentic virus neutralization results between control, homologous, and heterologous booster groups against **a** SARS-CoV-2 prototype strain. **b** Beta strain. **c** Gamma strain. **d** Delta strain. Horizontal bars denote geometric mean titers and according 95% credible. HCS stands for human convalescent serum. Booster doses were administrated on Day 0.

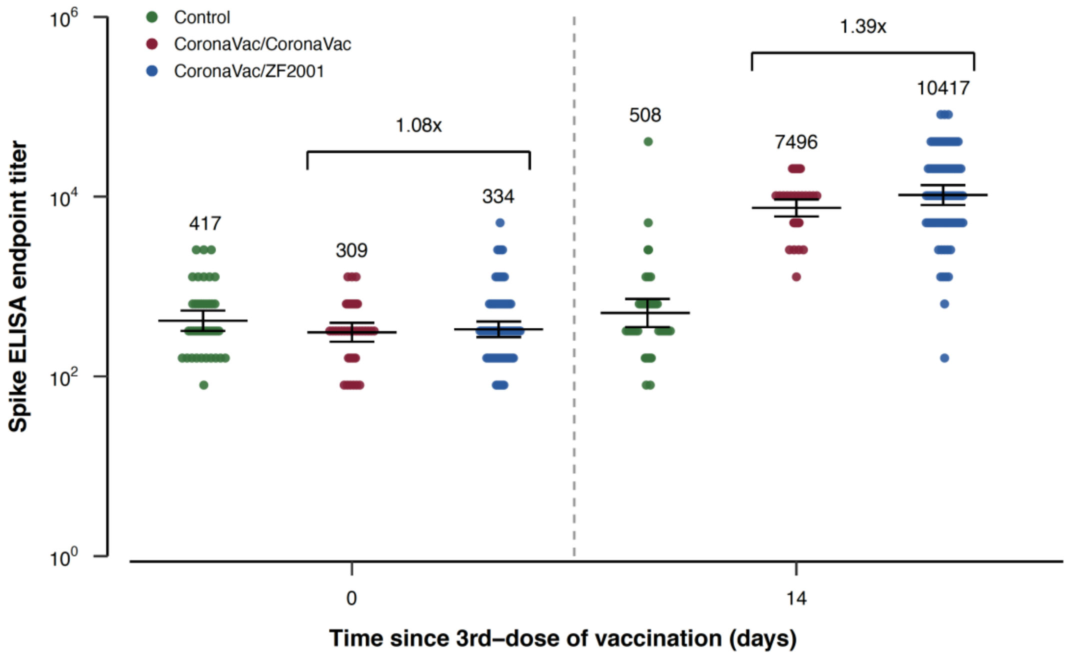

Fig. S3. SARS-CoV-2 anti-spike IgG antibody titer for all participants.

Comparison of anti-Spike IgG ELISA endpoint titers of plasma for each group. Horizontal bars denote geometric mean titers and according 95% credible interval.

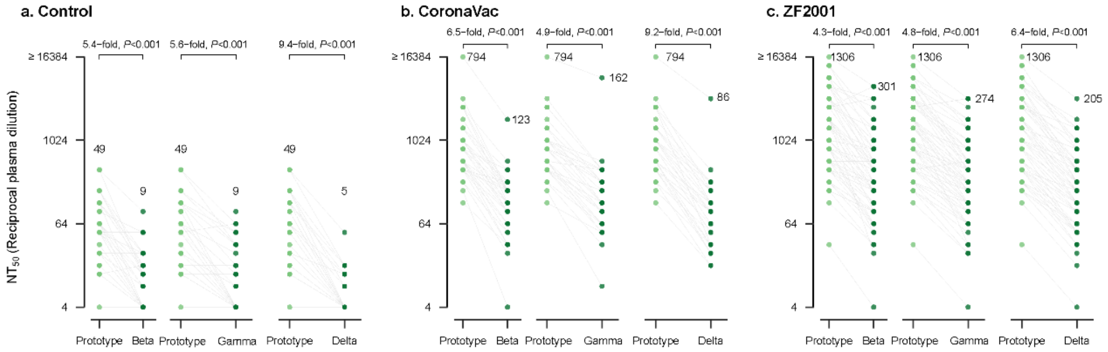

Fig. S4. Comparing neutralization level on day 14 between prototype and variants of concern

Authentic virus neutralization results against SARS-CoV-2 prototype and variants of concerns 14 days after boosting with **a** Control. **b** CoronaVac. **c** ZF2001. The geometric mean titer and pairwise fold-changes of 50% neutralization titers are labeled. Statistical significances were conducted by two-sided Wilcoxon signed-rank tests.

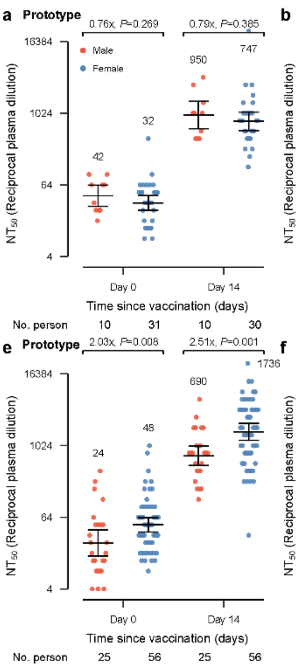

Fig. S5. Gender- and strain-specific humoral immune responses induced by third doses of CoronaVac or ZF2001

Comparison of humoral immune response differences between gender groups. **a-d** Neuralization antibody titer against prototype, Beta, Gamma, and Delta variants of SARS-CoV-2 after in the CoronaVac group; **e-h** Neutralization antibody titer against prototype, Beta, Gamma, and Delta variants in the ZF2001 group. Horizontal bars denote geometric mean titers and according 95% credible interval. Statistical significances were conducted by two-sided Wilcoxon rank-sum tests.

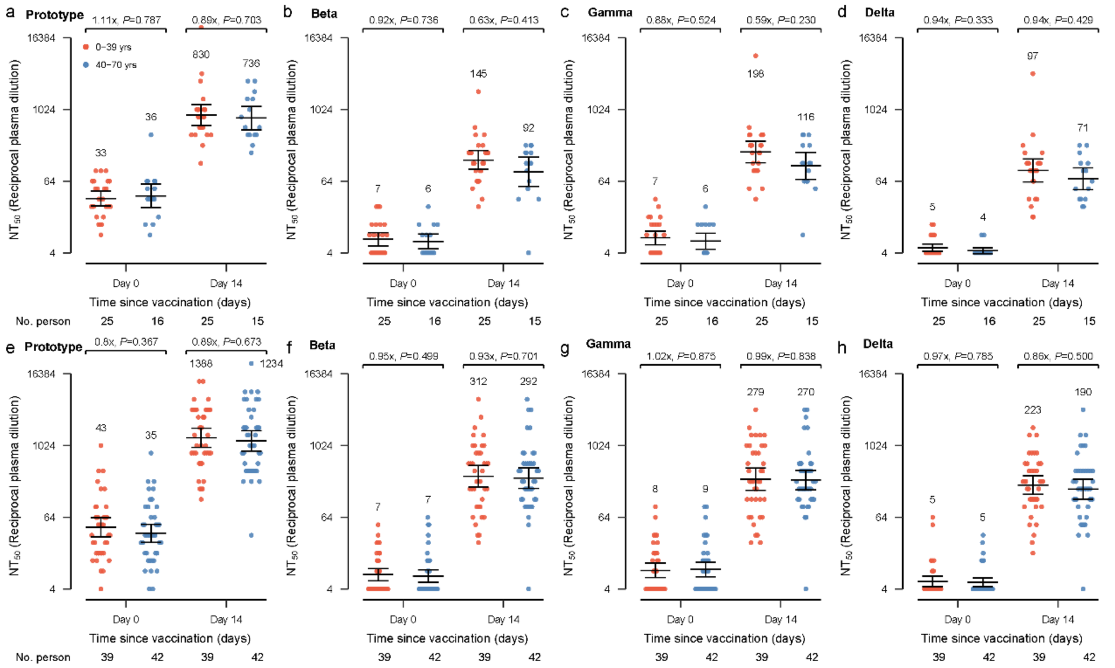

Fig. S6. Age- and strain-specific humoral immune responses induced by third doses of CoronaVac or ZF2001

Comparison of humoral immune response differences between age groups. **a-d** Neuralization antibody titer against prototype, Beta, Gamma, and Delta variants of SARS-CoV-2 after in the CoronaVac group; **e-h** Neutralization antibody titer against prototype, Beta, Gamma, and Delta variants in the ZF2001 group. Horizontal bars denote geometric mean titers and according 95% credible interval. Statistical significances were conducted by two-sided Wilcoxon rank-sum tests.

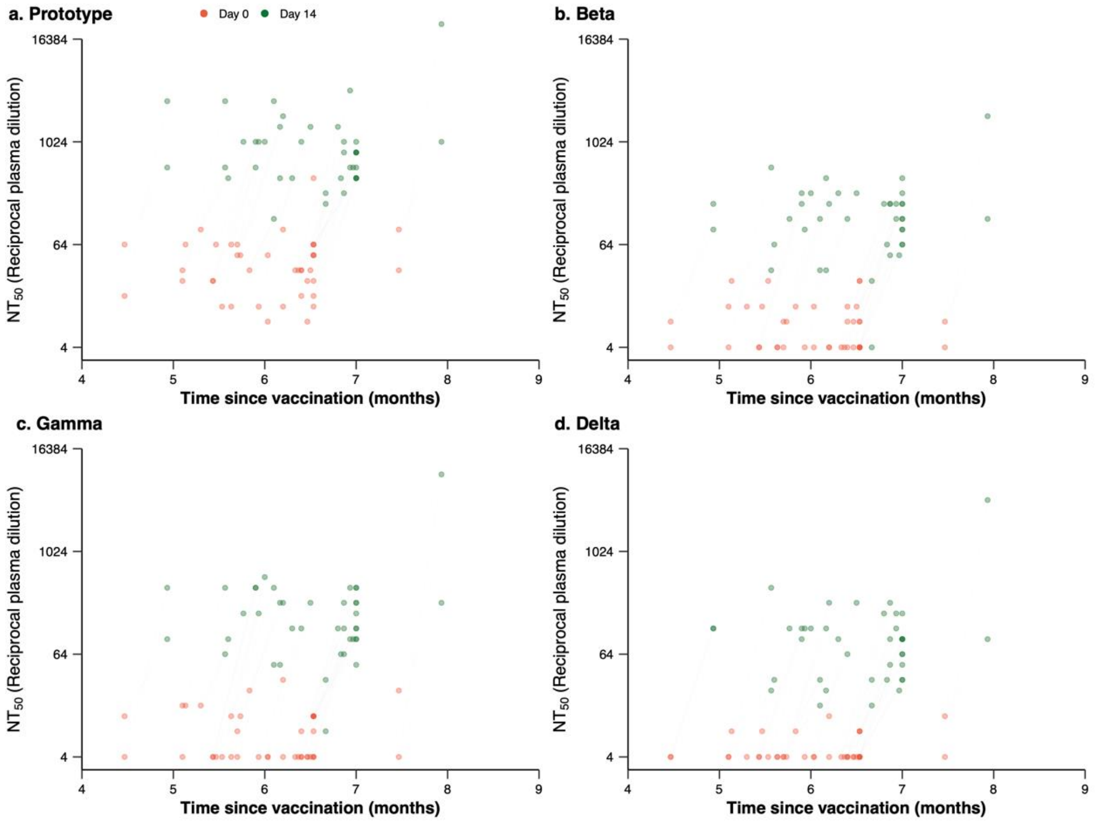

Fig. S7. Individual-level neutralization antibody titer against authentic SARS-CoV-2 in participants in the CoronaVac group after the second dose of CoronaVac

Distribution of the neutralizing titers for the homologous booster group based on the interval between primary immunization and the third dose. **a** Neutralizing titers against SARS-CoV-2 prototype strain. **b** Beta strain. **c** Gamma strain. **d** Delta strain. Booster doses were administrated on Day 0.

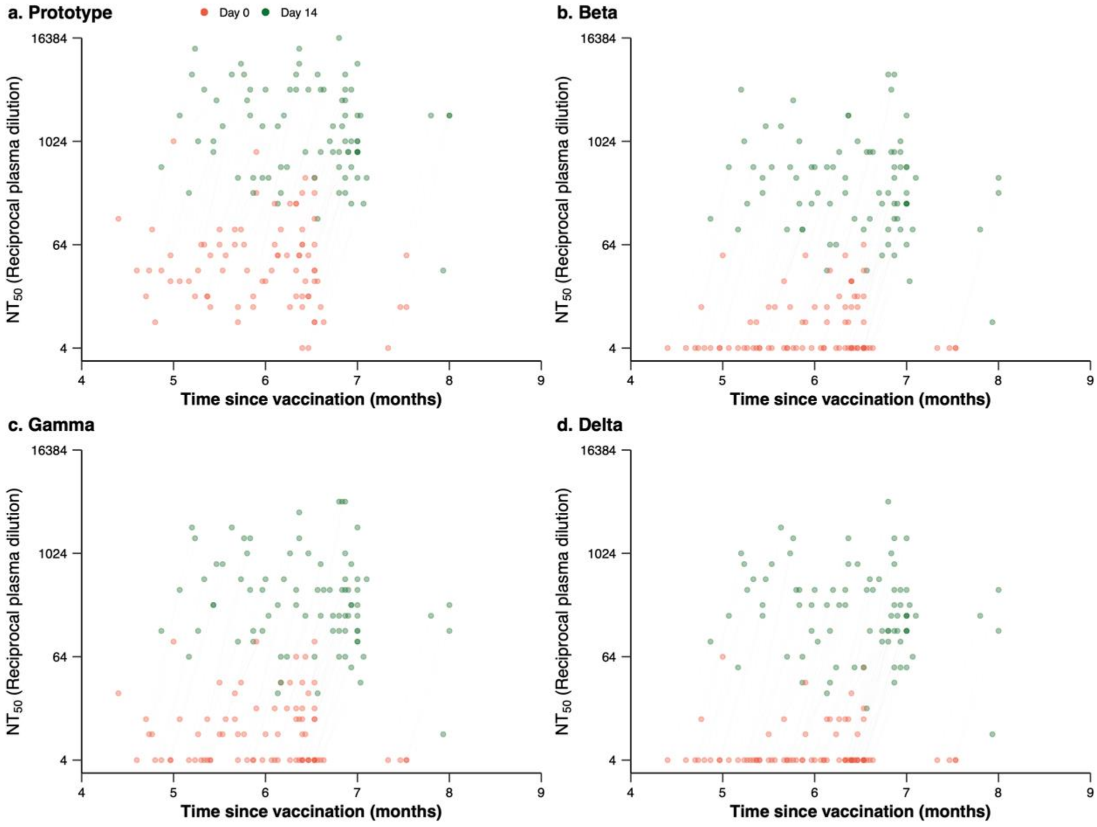

Fig. S8. Individual-level neutralization antibody titer against authentic SARS-CoV-2 in participants in the ZF2001 group after the second dose of CoronaVac

Distribution of the neutralizing titers for the heterologous booster group based on the interval between primary immunization and the third dose. **a** Neutralizing titers against SARS-CoV-2 prototype strain. **b** Beta strain. **c** Gamma strain. **d** Delta strain. Booster doses were administrated on Day 0.

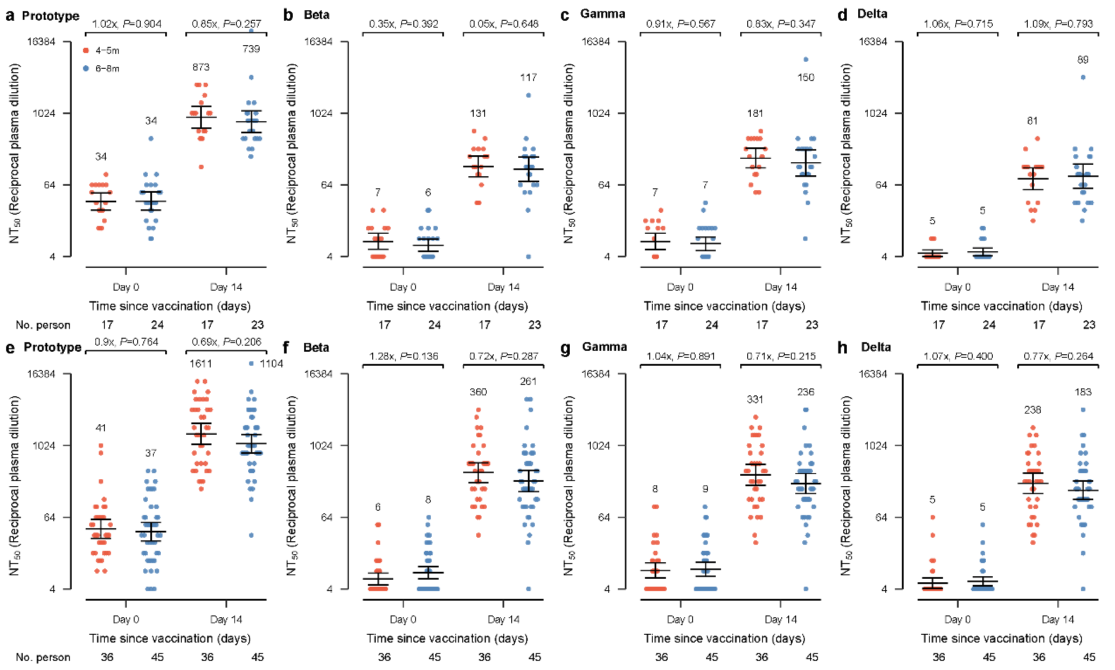

Fig. S9. Impact of the interval between primary immunization and the third doses of CoronaVac or ZF2001 on humoral immune responses

Comparison of humoral immune responses between groups with different intervals between primary immunization and the third dose. **a-d** Neuralization antibody titer against prototype, Beta, Gamma, and Delta variants of SARS-CoV-2 in participants administered with third doses in the CoronaVac group; **e-h** Neutralization antibody titer against prototype, Beta, Gamma, and Delta variants of SARS-CoV-2 in participants administered with third doses in the ZF2001 group. Horizontal bars denote geometric mean titers and according 95% credible interval. Statistical significances were conducted by two-sided Wilcoxon rank-sum tests.
